# Supplementary material for: Dissociation dynamics of the diamondoid adamantane upon photoionization by XUV femtosecond pulses
Source: Sci Rep. 2020 Feb 19;10:2884. doi: 10.1038/s41598-020-59649-1 (PMC7031298; doi:10.1038/s41598-020-59649-1)
Supplement: Supplementary file 1 — Supplementary Information. [file 41598_2020_59649_MOESM1_ESM.pdf]

Dissociation dynamics of the diamondoid adamantane upon photoionization by XUV femtosecond pulses

Supplementary Material

Sylvain Maclot[1,2,*], Jan Lahl[1], Jasper Peschel[1], Hampus Wikmark[1], Piotr Rudawski[1], Fabian Brunner[1], Hélène Coudert-Alteirac[1], Suvasthika Indrajith[3], Bernd A. Huber[3], Sergio Díaz-Tendero[4,5,6], Néstor F. Aguirre[7], Patrick Rousseau[3], and Per Johnsson[1,*]

[1]Department of Physics, Lund University, P.O. Box 118, 22100 Lund, Sweden
[2]Biomedical and X-Ray Physics, Department of Applied Physics, AlbaNova University Center, KTH Royal Institute of Technology, SE-10691 Stockholm, Sweden
[3]Normandie Université, ENSICAEN, UNICAEN, CEA, CNRS, CIMAP, 14000 Caen, France
[4]Departamento de Química, Módulo 13, Universidad Autónoma de Madrid, 28049 Madrid, Spain
[5]Condensed Matter Physics Center (IFIMAC), Universidad Autónoma de Madrid, 28049 Madrid, Spain
[6]Institute for Advanced Research in Chemical Sciences (IAdChem), Universidad Autónoma de Madrid, 28049 Madrid, Spain
[7]Theoretical Division, Los Alamos National Laboratory, Los Alamos, New Mexico 87545, USA
[*]smaclot@gmail.com and per.johnsson@fysik.lth.se

Contents

1 Total ion VMI **2**

2 Excited states of adamantane dication **3**

3 Survival of adamantane dication **4**

4 Molecular systems used in this work **5**

H, H_2, H_3C, H_4C, H_4C_2 . . . 6
H_4C_3, H_6C_2, H_5C_3, H_7C_3, H_6C_4 . . . 6
H^+, H_8C_3, H_6C_6, H_3C^+, H_8C_6 . . . 7
H_8C_7, $H_5C_2^+$, $H_5C_3^+$, $H_6C_3^+$, $H_7C_3^+$ . . . 8
$H_6C_4^+$, $H_7C_4^+$, $H_6C_5^+$, $H_7C_5^+$, $H_6C_6^+$ . . . 9
$H_7C_6^+$, $H_8C_6^+$, $H_7C_7^+$, $H_9C_6^+$, $H_8C_7^+$ . . . 10
$H_9C_7^+$, $H_{10}C_7^+$, $H_9C_8^+$ . . . 11
$H_{11}C_7^+$, $H_{11}C_8^+$ . . . 12
$H_{16}C_{10}^{2+}$ . . . 13

1 Total ion VMI

The figure 1 shows the total ion VMI signal obtained after the 275000 laser shots. The center of the detector, so of the image, is located at (500 px, 500 px). Due to the velocity of the supersonic jet ($\sim 1000\,\mathrm{m\cdot s^{-1}}$) coming from top of this image (perpendicular to the detector axis) we observe different features corresponding to different ionic species (different masses) distributed along the vertical axis from the center towards the bottom. The intense signal close to the center correspond to helium and the second most intense signal to the parent ion. Those signals are so intense compare to the rest that some of it cannot be completely removed while applying covariance analysis leading to artefacts visible in the panels (a) and (b) of figure 7 in the main article.

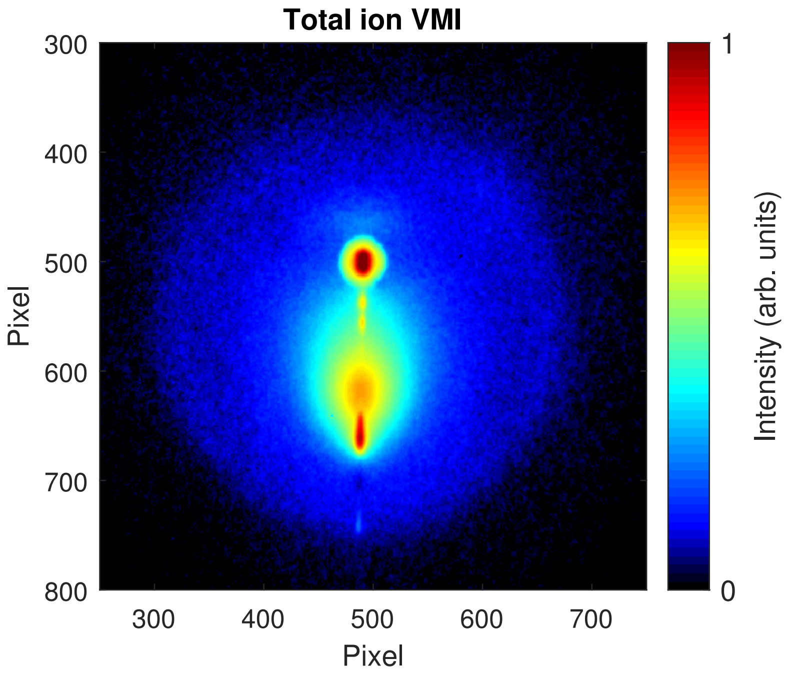

Figure 1. Total ion VMI signal (log scale).

2 Excited states of adamantane dication

We have perfomed time-dependent density functional theory (TDDFT) simulations to compute the electronic excited states populated in the photoionization. To this we have used the geometry of the neutral adamantane, doubly ionized. We have chosen the CAM-B3LYP functional in combination with the 6-311G* atomic basis set. The first 300 excited states have been obtained for the adamantane dication (assuming a Franck-Condon ionization). Computed excitation energies and oscillator strengths are given in Figure 2. The energy of the states is given relative to the neutral ground state, i.e. shifted by the second ionization threshold.

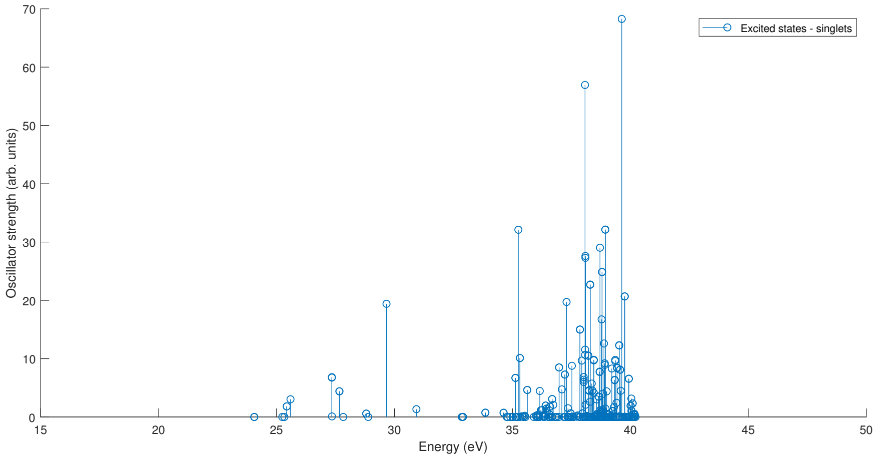

Figure 2. 300 first singlet excited states of the adamantane dication.

3 Survival of adamantane dication

Using molecular dynamics simulations within the density functional tight binding (DFTB) method, we have computed the survival time of the doubly ionized adamantane molecule at different excitation energies. Molecular reorganization takes place (leading mainly to the open cage structures shown in the main article), but fragmentation appears typically after a few hundred of picoseconds with the lowest excitation energy values here considered.

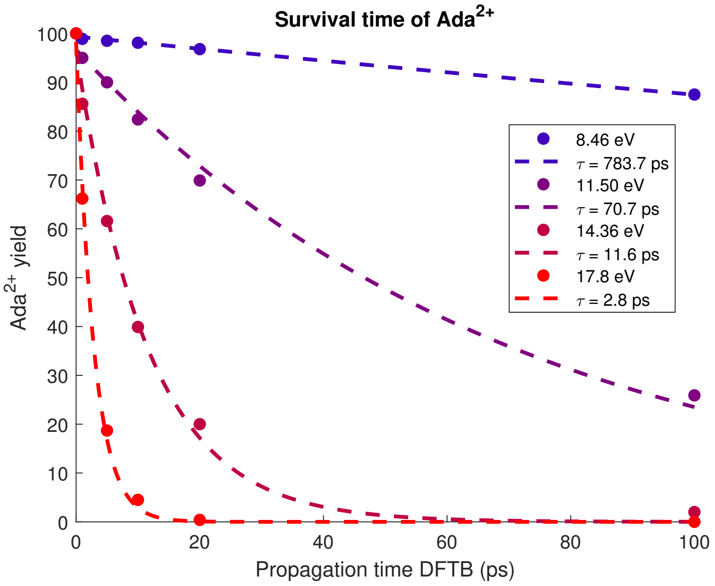

Figure 3. Yield of intact adamantane dication as function of propagation time of the molecular dynamics calculations for 4 excitation energies 8.46, 11.50, 14.35 and 17.80 eV, corresponding to the harmonic orders 21, 23, 25 and 27 respectively. Level of theory DFTB for 1000 trajectories.

4 Molecular systems used in this work

The next pages show the structure, relative energy (in eV), file name and symmetry of all the stable molecules used in this work. The file names have the following format:

`<stoichiometry>.q<charge>.m<multiplicity>-<id>`

To illustrate the `.rxyz` which is the standard format used by M3C, information of the lowest energy state of the H_3C molecule is shown below. This format basically follows the same format as the traditional `.xyz` files. Line 1: number of atoms n, line 2: comment (it includes the value of the energy in atomic units), lines from 3 up to $3+n$: symbols and atomic positions in Å). Additionally, lines 8-15: calculated vibrational frequencies in cm^{-1}, line 17: symmetry group of the molecule, and line 18: the symmetry of the wave function. All structures in the `.rxyz` format are also available as a supplementary file (`geometries-adamantane.zip`)

```
1  4
2  Energy =   -39.8382921987
3      C    1.847874    1.221505   -0.218795
4      H    2.358454    0.319387    0.094162
5      H    2.358473    1.943584   -0.843569
6      H    0.827159    1.402204    0.094162
7
8  FREQUENCIES 6
9  453.5368
10 1431.1588
11 1431.1605
12 3143.2894
13 3317.9043
14 3317.9052
15
16 SYMMETRY C3V
17 ELECTRONIC_STATE 2-A1
```

H

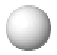

1
H.q0.m2-1
2-S (R3)
0.00 eV

H_2

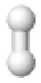

1
H2.q0.m1-1
1-SGG (D*H)
0.00 eV

H_3C

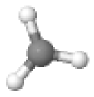

1
H3C.q0.m2-1
2-A1 (C3V)
0.00 eV

H_4C

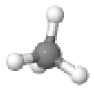

1
H4C.q0.m1-1
1-A (C1)
0.00 eV

H_4C_2

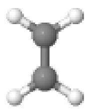

1
H4C2.q0.m1-1
1-A' (CS)
0.00 eV

H_4C_3

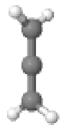

1
H4C3.q0.m1-1
1-A (C1)
0.00 eV

H_6C_2

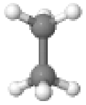

1
H6C2.q0.m1-1
1-A1 (D3)
0.00 eV

H_5C_3

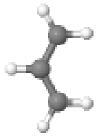

1
H5C3.q0.m2-1
2-A (C1)
0.00 eV

H_7C_3

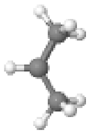

1
H7C3.q0.m2-1
2-A (C1)
0.00 eV

H_6C_4

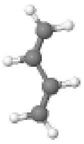

1
H6C4.q0.m1-1
1-A' (CS)
0.00 eV

H^+

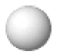
1<br>H.q1.m1-1<br>1-S (R3)<br>0.00 eV

H_8C_3

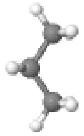
1<br>H8C3.q0.m1-1<br>1-A (C1)<br>0.00 eV

H_6C_6

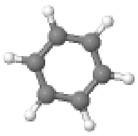	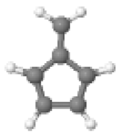	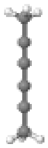
1<br>H6C6.q0.m1-1<br>1-A (C1)<br>0.00 eV	2<br>H6C6.q0.m1-3<br>1-A (C1)<br>1.56 eV	3<br>H6C6.q0.m1-2<br>1-A1 (D3)<br>3.02 eV

H_3C^+

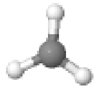
1<br>H3C.q1.m1-1<br>1-A1 (C3V)<br>0.00 eV

H_8C_6

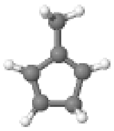	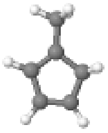	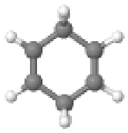	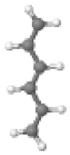	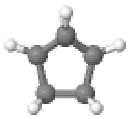
1<br>H8C6.q0.m1-3<br>1-A (C1)<br>0.00 eV	2<br>H8C6.q0.m1-4<br>1-A (C1)<br>0.01 eV	3<br>H8C6.q0.m1-2<br>1-A (C1)<br>0.08 eV	4<br>H8C6.q0.m1-5<br>1-A' (CS)<br>0.63 eV	5<br>H8C6.q0.m1-1<br>1-A (C1)<br>1.44 eV

H_8C_7		
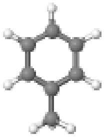<br>1<br>H8C7.q0.m1-1<br>1-A (C1)<br>0.00 eV	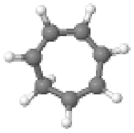<br>2<br>H8C7.q0.m1-2<br>1-A (C1)<br>1.55 eV	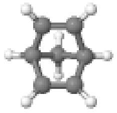<br>3<br>H8C7.q0.m1-3<br>1-A (C1)<br>2.43 eV

$H_5C_2^+$
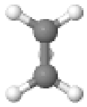<br>1<br>H5C2.q1.m1-1<br>1-A (C1)<br>0.00 eV

$H_5C_3^+$
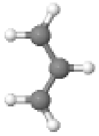<br>1<br>H5C3.q1.m1-1<br>1-A (C1)<br>0.00 eV

$H_6C_3^+$	
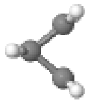<br>1<br>H6C3.q1.m2-3<br>2-A (C1)<br>0.00 eV	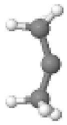<br>2<br>H6C3.q1.m2-2<br>2-A (C1)<br>0.33 eV

$H_7C_3^+$
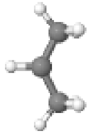<br>1<br>H7C3.q1.m1-1<br>1-A (C1)<br>0.00 eV

$H_6C_4^+$
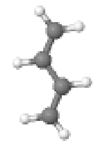<br>1<br>H6C4.q1.m2-1<br>2-A" (CS)<br>0.00 eV

$H_7C_4^+$
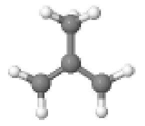<br>1<br>H7C4.q1.m1-1<br>1-A (C1)<br>0.00 eV

$H_6C_5^+$				
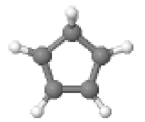<br>1<br>H6C5.q1.m2-1<br>2-A (C1)<br>0.00 eV	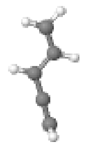<br>2<br>H6C5.q1.m2-5<br>2-A (C1)<br>1.31 eV	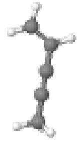<br>3<br>H6C5.q1.m2-4<br>2-A (C1)<br>1.46 eV	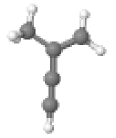<br>4<br>H6C5.q1.m2-2<br>2-A (C1)<br>1.93 eV	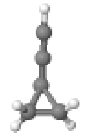<br>5<br>H6C5.q1.m2-3<br>2-A (C1)<br>2.39 eV

$H_7C_5^+$			
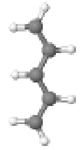<br>1<br>H7C5.q1.m1-1<br>1-A (C1)<br>0.00 eV	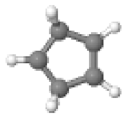<br>2<br>H7C5.q1.m1-3<br>1-A (C1)<br>0.48 eV	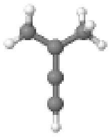<br>3<br>H7C5.q1.m1-2<br>1-A (C1)<br>1.04 eV	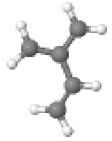<br>4<br>H7C5.q1.m1-4<br>1-A (C1)<br>1.11 eV

$H_6C_6^+$		
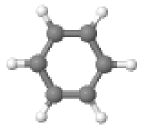<br>1<br>H6C6.q1.m2-1<br>2-A (C1)<br>0.00 eV	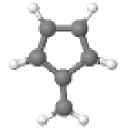<br>2<br>H6C6.q1.m2-3<br>2-A (C1)<br>0.52 eV	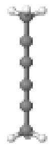<br>3<br>H6C6.q1.m2-2<br>2-A (C1)<br>2.45 eV

$H_7C_6^+$			
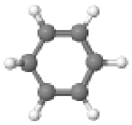	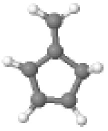	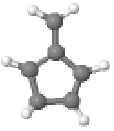	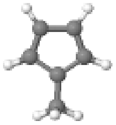
1<br>H7C6.q1.m1-2<br>1-A (C1)<br>0.00 eV	2<br>H7C6.q1.m1-3<br>1-A (C1)<br>0.43 eV	3<br>H7C6.q1.m1-4<br>1-A (C1)<br>1.21 eV	4<br>H7C6.q1.m1-1<br>1-A (C1)<br>1.53 eV

$H_8C_6^+$				
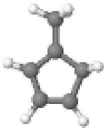	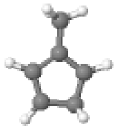	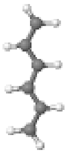	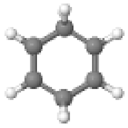	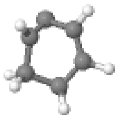
1<br>H8C6.q1.m2-4<br>2-A (C1)<br>0.00 eV	2<br>H8C6.q1.m2-3<br>2-A (C1)<br>0.13 eV	3<br>H8C6.q1.m2-5<br>2-A" (CS)<br>0.60 eV	4<br>H8C6.q1.m2-2<br>2-A (C1)<br>0.71 eV	5<br>H8C6.q1.m2-1<br>2-A (C1)<br>2.43 eV

$H_7C_7^+$				
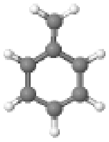	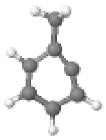	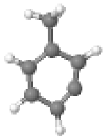	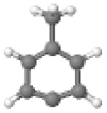	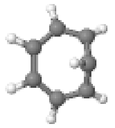
1<br>H7C7.q1.m1-2<br>1-A (C1)<br>0.00 eV	2<br>H7C7.q1.m1-5<br>1-A (C1)<br>1.91 eV	3<br>H7C7.q1.m1-4<br>1-A (C1)<br>2.00 eV	4<br>H7C7.q1.m1-3<br>1-A (C1)<br>2.09 eV	5<br>H7C7.q1.m1-1<br>1-A (C1)<br>2.27 eV

$H_9C_6^+$	
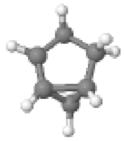	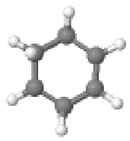
1<br>H9C6.q1.m1-1<br>1-A (C1)<br>0.00 eV	2<br>H9C6.q1.m1-2<br>1-A (C1)<br>0.37 eV

$H_8C_7^+$		
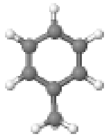	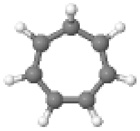	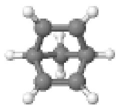
1<br>H8C7.q1.m2-1<br>2-A (C1)<br>0.00 eV	2<br>H8C7.q1.m2-2<br>2-A (C1)<br>0.61 eV	3<br>H8C7.q1.m2-3<br>2-A (C1)<br>1.83 eV

$H_9C_7^+$				
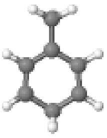<br>1<br>H9C7.q1.m1-5<br>1-A (C1)<br>0.00 eV	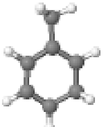<br>2<br>H9C7.q1.m1-3<br>1-A (C1)<br>0.21 eV	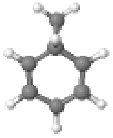<br>3<br>H9C7.q1.m1-4<br>1-A (C1)<br>0.40 eV	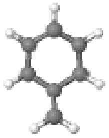<br>4<br>H9C7.q1.m1-6<br>1-A (C1)<br>1.26 eV	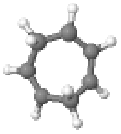<br>5<br>H9C7.q1.m1-2<br>1-A (C1)<br>1.41 eV
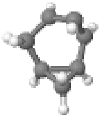<br>6<br>H9C7.q1.m1-1<br>1-A (C1)<br>3.51 eV				

$H_{10}C_7^+$				
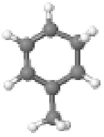<br>1<br>H10C7.q1.m2-9<br>2-A (C1)<br>0.00 eV	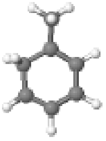<br>2<br>H10C7.q1.m2-1<br>2-A (C1)<br>0.41 eV	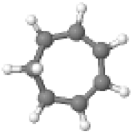<br>3<br>H10C7.q1.m2-4<br>2-A (C1)<br>0.54 eV	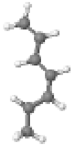<br>4<br>H10C7.q1.m2-7<br>2-A (C1)<br>0.64 eV	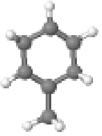<br>5<br>H10C7.q1.m2-6<br>2-A (C1)<br>0.70 eV
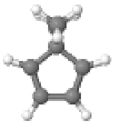<br>6<br>H10C7.q1.m2-2<br>2-A (C1)<br>0.73 eV	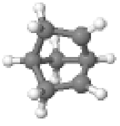<br>7<br>H10C7.q1.m2-5<br>2-A (C1)<br>1.26 eV	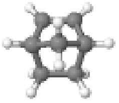<br>8<br>H10C7.q1.m2-3<br>2-A (C1)<br>1.33 eV	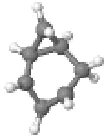<br>9<br>H10C7.q1.m2-10<br>2-A (C1)<br>2.09 eV	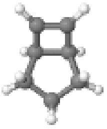<br>10<br>H10C7.q1.m2-8<br>2-A (C1)<br>2.18 eV

$H_9C_8^+$			
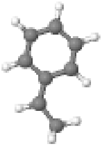<br>1<br>H9C8.q1.m1-1<br>1-A (C1)<br>0.00 eV	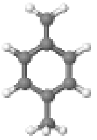<br>2<br>H9C8.q1.m1-3<br>1-A (C1)<br>0.13 eV	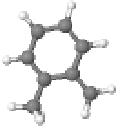<br>3<br>H9C8.q1.m1-2<br>1-A (C1)<br>0.28 eV	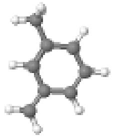<br>4<br>H9C8.q1.m1-4<br>1-A (C1)<br>0.30 eV

$H_{11}C_7^+$	
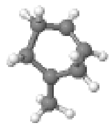<br>1<br>H11C7.q1.m1-2<br>1-A (C1)<br>0.00 eV	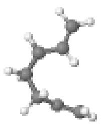<br>2<br>H11C7.q1.m1-1<br>1-A (C1)<br>1.13 eV

$H_{11}C_8^+$				
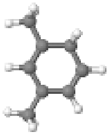<br>1<br>H11C8.q1.m1-7<br>1-A (C1)<br>0.00 eV	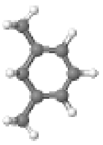<br>2<br>H11C8.q1.m1-9<br>1-A (C1)<br>0.05 eV	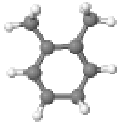<br>3<br>H11C8.q1.m1-3<br>1-A (C1)<br>0.18 eV	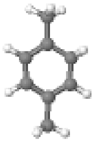<br>4<br>H11C8.q1.m1-5<br>1-A (C1)<br>0.20 eV	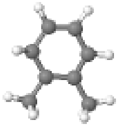<br>5<br>H11C8.q1.m1-10<br>1-A (C1)<br>0.24 eV
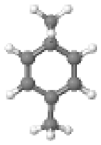<br>6<br>H11C8.q1.m1-6<br>1-A (C1)<br>0.33 eV	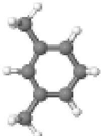<br>7<br>H11C8.q1.m1-4<br>1-A (C1)<br>0.35 eV	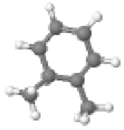<br>8<br>H11C8.q1.m1-1<br>1-A (C1)<br>0.41 eV	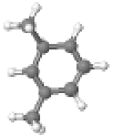<br>9<br>H11C8.q1.m1-8<br>1-A (C1)<br>0.54 eV	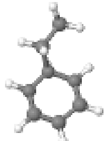<br>10<br>H11C8.q1.m1-2<br>1-A (C1)<br>0.74 eV
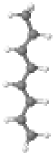<br>11<br>H11C8.q1.m1-24<br>1-A (C1)<br>1.00 eV	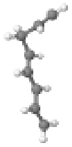<br>12<br>H11C8.q1.m1-19<br>1-A (C1)<br>1.91 eV	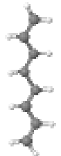<br>13<br>H11C8.q1.m1-20<br>1-A (C1)<br>2.63 eV		

$H_{16}C_{10}^{2+}$				
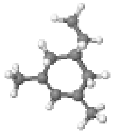<br>1<br>H16C10.q2.m1-158<br>1-A (C1)<br>0.00 eV	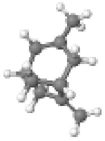<br>2<br>H16C10.q2.m1-31<br>1-A (C1)<br>0.05 eV	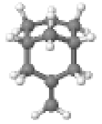<br>3<br>H16C10.q2.m1-9<br>1-A (C1)<br>0.28 eV	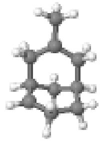<br>4<br>H16C10.q2.m1-78<br>1-A (C1)<br>0.28 eV	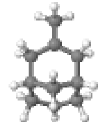<br>5<br>H16C10.q2.m1-85<br>1-A (C1)<br>0.32 eV
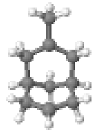<br>6<br>H16C10.q2.m1-10<br>1-A (C1)<br>0.32 eV	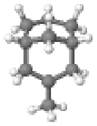<br>7<br>H16C10.q2.m1-54<br>1-A (C1)<br>0.32 eV	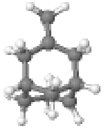<br>8<br>H16C10.q2.m1-88<br>1-A (C1)<br>0.46 eV	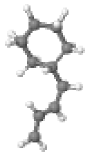<br>9<br>H16C10.q2.m1-119<br>1-A (C1)<br>0.48 eV	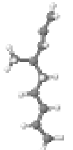<br>10<br>H16C10.q2.m1-12<br>1-A (C1)<br>0.53 eV
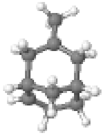<br>11<br>H16C10.q2.m1-73<br>1-A (C1)<br>0.60 eV	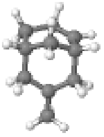<br>12<br>H16C10.q2.m1-99<br>1-A (C1)<br>0.60 eV	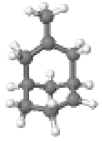<br>13<br>H16C10.q2.m1-140<br>1-A (C1)<br>0.60 eV	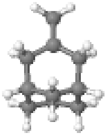<br>14<br>H16C10.q2.m1-105<br>1-A (C1)<br>0.60 eV	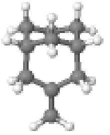<br>15<br>H16C10.q2.m1-4<br>1-A (C1)<br>0.60 eV
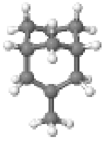<br>16<br>H16C10.q2.m1-92<br>1-A (C1)<br>0.61 eV	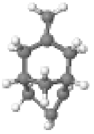<br>17<br>H16C10.q2.m1-97<br>1-A (C1)<br>0.63 eV	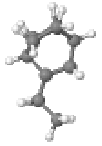<br>18<br>H16C10.q2.m1-152<br>1-A (C1)<br>0.67 eV	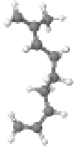<br>19<br>H16C10.q2.m1-126<br>1-A (C1)<br>0.67 eV	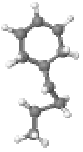<br>20<br>H16C10.q2.m1-160<br>1-A (C1)<br>0.69 eV
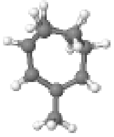<br>21<br>H16C10.q2.m1-133<br>1-A (C1)<br>0.70 eV	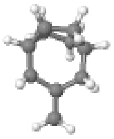<br>22<br>H16C10.q2.m1-67<br>1-A (C1)<br>0.70 eV	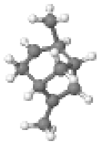<br>23<br>H16C10.q2.m1-128<br>1-A (C1)<br>0.71 eV	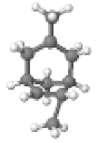<br>24<br>H16C10.q2.m1-14<br>1-A (C1)<br>0.71 eV	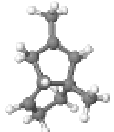<br>25<br>H16C10.q2.m1-63<br>1-A (C1)<br>0.73 eV
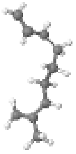<br>26<br>H16C10.q2.m1-114<br>1-A (C1)<br>0.75 eV	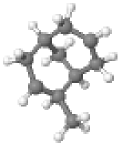<br>27<br>H16C10.q2.m1-59<br>1-A (C1)<br>0.99 eV	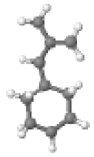<br>28<br>H16C10.q2.m1-60<br>1-A (C1)<br>1.01 eV	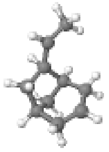<br>29<br>H16C10.q2.m1-6<br>1-A (C1)<br>1.06 eV	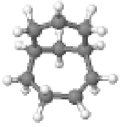<br>30<br>H16C10.q2.m1-96<br>1-A (C1)<br>1.07 eV

$H_{16}C_{10}^{2+}$ (*Continued from previous page*)				
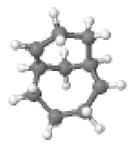<br>31<br>H16C10.q2.m1-32<br>1-A (C1)<br>1.12 eV	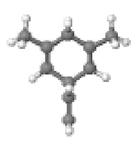<br>32<br>H16C10.q2.m1-52<br>1-A (C1)<br>1.12 eV	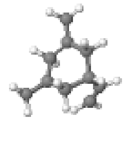<br>33<br>H16C10.q2.m1-123<br>1-A (C1)<br>1.22 eV	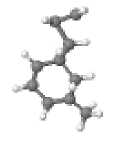<br>34<br>H16C10.q2.m1-15<br>1-A (C1)<br>1.23 eV	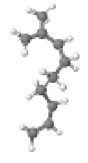<br>35<br>H16C10.q2.m1-127<br>1-A (C1)<br>1.23 eV
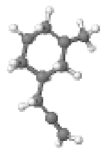<br>36<br>H16C10.q2.m1-28<br>1-A (C1)<br>1.24 eV	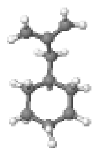<br>37<br>H16C10.q2.m1-44<br>1-A (C1)<br>1.25 eV	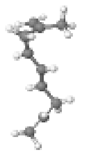<br>38<br>H16C10.q2.m1-33<br>1-A (C1)<br>1.26 eV	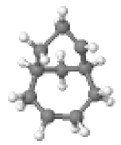<br>39<br>H16C10.q2.m1-61<br>1-A (C1)<br>1.30 eV	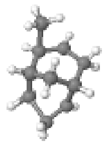<br>40<br>H16C10.q2.m1-89<br>1-A (C1)<br>1.30 eV
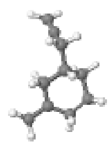<br>41<br>H16C10.q2.m1-7<br>1-A (C1)<br>1.31 eV	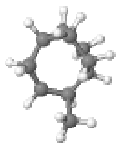<br>42<br>H16C10.q2.m1-41<br>1-A (C1)<br>1.39 eV	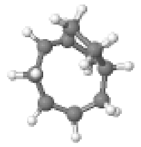<br>43<br>H16C10.q2.m1-149<br>1-A (C1)<br>1.41 eV	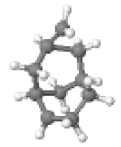<br>44<br>H16C10.q2.m1-51<br>1-A (C1)<br>1.47 eV	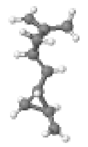<br>45<br>H16C10.q2.m1-64<br>1-A (C1)<br>1.57 eV
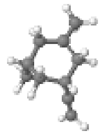<br>46<br>H16C10.q2.m1-37<br>1-A (C1)<br>1.65 eV	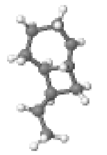<br>47<br>H16C10.q2.m1-47<br>1-A (C1)<br>1.71 eV	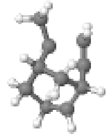<br>48<br>H16C10.q2.m1-25<br>1-A (C1)<br>1.73 eV	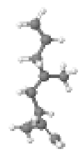<br>49<br>H16C10.q2.m1-146<br>1-A (C1)<br>1.91 eV	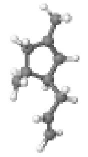<br>50<br>H16C10.q2.m1-138<br>1-A (C1)<br>2.04 eV
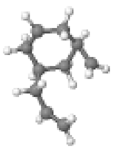<br>51<br>H16C10.q2.m1-74<br>1-A (C1)<br>2.68 eV				
